# Laundry Accessories

**Published:** 1912-11-16

**Authors:** 


### Laundry Accessories.

### ANOTHER SOAP POWDER.

Simon's Lessive Soap Powder is disinfectant, and for this reason is very suitable for hospital laundry use. It contains nothing which will injure the linen, and can be used without admixture of further soap or soda.

### SOME RELIABLE SOAPS.

For general laundry work it is hard to beat Edward Cook and Son's Gold Medal Mottled Soap, which is both durable and effectual. The Genuine Brown Soap of the same makers is particularly to be recommended for washing flannels, as, being an oil soap, it renders the fabrics soft and supple, and its use under proper conditions obviates all danger of shrinkage or deterioration. Laundries which have not the advantage of a highly-trained manageress are often glad to dispense with the preliminary operations necessitated by the use of solid soaps, and for such we can cordially recommend Cook's Soap Powder. It can be used both for linen and flannel goods, and put direct into the outer cage, so that all difficulties are smoothed away.

### WATER SOFTENING AND CALORIFIERS.

Royles, Limited, has brought out a process for water-purification on Reisert's System which should be of benefit in many institutions situated in localities where the water is impregnated with lime. The bicarbonate of lime is precipitated by the use of hydrate of lime, and the sulphate of lime by soda. The softened water is then filtered and discharged. Another good appliance of this firm is Coker's Patent Vapouriser, for delivering pure boiler steam properly controlled and free from condensation and consequent drip. Managers in need of thoroughly satisfactory Calorifiers should not omit to obtain particulars of those manufactured by Messrs. Royles, Limited, fitted with Row's Patent Indented Tubes, and with Royle's Patent Automatic Steam Control, by means of which the water can be controlled at any desired temperature. It is certainly an immense advantage for the water for baths to be maintained at the same temperature during any time of the day or night.

